# GSH-Triggered/Photothermal-Enhanced H_2_S Signaling Molecule Release for Gas Therapy

**DOI:** 10.3390/pharmaceutics15102443

**Published:** 2023-10-10

**Authors:** Xinqiang Liang, Mekhrdod S. Kurboniyon, Yuanhan Zou, Kezong Luo, Shuhong Fang, Pengle Xia, Shufang Ning, Litu Zhang, Chen Wang

**Affiliations:** 1 Department of Research, Guangxi Cancer Molecular Medicine Engineering Research Center, Guangxi Medical University Cancer Hospital, Nanning 530021, China; xx03716@163.com (X.L.); 20205010739@sr.gxmu.edu.cn (Y.Z.); 20205010735@sr.gxmu.edu.cn (K.L.); 20205010724@sr.gxmu.edu.cn (S.F.); 20205010708@sr.gxmu.edu.cn (P.X.); ningshufang@gxmu.edu.cn (S.N.); 2 National Academy of Sciences of Tajikistan, Dushanbe 734000, Tajikistan; mehrdodq@gmail.com

**Keywords:** gas therapy, H_2_S, GSH-triggered, Prussian blue, dendritic mesoporous organosilica

## Abstract

Traditional treatment methods for tumors are inefficient and have severe side effects. At present, new therapeutic methods such as phototherapy, chemodynamic therapy, and gasodynamic therapy have been innovatively developed. High concentrations of hydrogen sulfide (H_2_S) gas exhibit cancer-suppressive effects. Herein, a Prussian blue-loaded tetra-sulfide modified dendritic mesoporous organosilica (PB@DMOS) was rationally constructed with glutathione (GSH)-triggered/photothermal-enhanced H_2_S signaling molecule release properties for gas therapy. The as-synthesized nanoplatform confined PB nanoparticles in the mesoporous structure of organosilica silica due to electrostatic adsorption. In the case of a GSH overexpressed tumor microenvironment, H_2_S gas was controllably released. And the temperature increases due to the photothermal effects of PB nanoparticles, further enhancing H_2_S release. At the same time, PB nanoparticles with excellent hydrogen peroxide catalytic performance also amplified the efficiency of tumor therapy. Thus, a collective nanoplatform with gas therapy/photothermal therapy/catalytic therapy functionalities shows potential promise in terms of efficient tumor therapy.

## 1. Introduction

Cancer is still a worldwide issue that needs to be urgently solved [1]. Traditional methods of tumor treatment include surgical resection, radiation therapy, and chemotherapy [2,3,4]. However, due to contraindications, side effects, and poor specificity, traditional treatments cannot achieve the expected effect and cannot effectively improve the quality of life of patients [5,6,7,8,9]. Chemotherapy, for example, uses toxic compounds and drugs to destroy tumor cells by preventing them from growing and dividing further [10,11,12]. Tumor cells divide and grow much faster than normal cells, resulting in very high doses of drugs required for treatment, which can cause a large number of normal cells to be killed. The body will develop resistance after long-term exposure to chemical drugs, which greatly reduces the therapeutic effect of chemotherapy. Half of cancer patients undergo radiation therapy to delay or cure the disease. Radiotherapy is the use of ionizing radiation to kill tumor cells, while the surrounding normal cells may also be damaged, so there are large side effects that can lead to adverse reactions in patients [13,14,15,16]. Moreover, radiotherapy can lead to short-term or long-term toxicity, where the long-term toxicity is irreversible and even gradually accumulates and increases, seriously affecting the health of patients. Therefore, we urgently need new therapeutic methods with strong specificity and small toxic side effects. According to the unique advantages, phototherapy, chemodynamic therapy, and gasodynamic therapy have been innovatively developed and show good advanced potential that is promising in terms tumor therapy [17,18,19,20,21,22,23,24,25,26,27].

Gas signaling molecules play a role in regulating physiological functions, which also have certain therapeutic effects on cancer [28,29,30,31]. Gas therapy is the use of cancer-suppressive gas molecules at high levels to kill tumor cells. Moderately high concentrations of these three gas molecules can reverse the Warburg effect in tumor cells by blocking their survival pathways [32]. Because gas molecules are difficult to control and measure accurately, gas therapy requires the delivery of gas-releasing molecules to the target site and then the control of gas release through endogenous or exogenous stimuli to guarantee the lowest systemic toxicity and targeted drug delivery [33,34,35]. Endogenous stimuli that have been studied include the tumor microenvironment, such as weak acid condition, high levels of glutathione (GSH), and overexpressed hydrogen peroxide (H_2_O_2_), which can reach deep tissues and are non-invasive. Exogenous stimuli, including ultrasound (UC), near-infrared light (NIR), microwaves (MV), magnetic forces (MF), and X-rays, can more precisely control the amount and timing of gas release.

Hydrogen sulfide (H_2_S) is the third gas signaling molecule discovered after nitric oxide (NO) [36,37] and (CO) [38]. Studies have found that the human body also generates endogenous H_2_S and participates in cell activities, such as autophagy, apoptosis, and proliferation, and is involved in regulating multiple signaling pathways, with antioxidant, anti-inflammatory, anti-tumor, and other activities [39,40,41,42]. Similar to NO and CO [43,44,45,46,47], H_2_S tends to play a role in inducing vasodilation, cell protection, and anti-inflammatory at low concentrations and shows a cancer-promoting effect in tumor cells. H_2_S exhibits a “bell model” in its effects on tumor cells that is, it exhibits a cancer-promoting effect at a relatively lower concentration and a cancer-inhibiting effect at a relatively higher concentration. When exposed to an environment with high H_2_S levels (generally given externally) or for a long time, the anti-proliferation effect can be exerted, and the vitality of cells can be damaged through various mechanisms such as mitochondrial inhibition, activation of apoptosis signal, and intracellular acidification. Solving the difficulty of how to accurately control the dose of the drug and realizing the transportation of the H_2_S gas in the treatment process are the keys. Fortunately, by combining the advantages of nanotechnology, the rational design of nanomaterials provides a possible strategy.

Herein, Prussian blue-loaded tetra-sulfide modified dendritic mesoporous organosilica (PB@DMOS) was rationally constructed GSH-triggered/photothermal-enhanced H_2_S signaling molecule release properties for gas therapy. The as-synthesized nanoplatform confined PB nanoparticles (NPs) in the mesoporous structure of organosilica silica electrostatic adsorption. GSH overexpressed tumor microenvironment, H_2_S gas was controllably released. The temperature increases due to thephotothermal effects of PB NPs further enhancing H_2_S release. At the same time, PB NPs with excellent H_2_O_2_ catalytic performance also amplified the efficiency of tumor therapy. Thus, a collective nanoplatform with gas therapy/photothermal therapy/catalytic therapy functionalities advanced promising in terms of efficient tumor treatment.

## 2. Materials and Methods

### 2.1. Chemical Reagents

Potassium ferricyanide (K_3_[Fe(CN)_6_], 99%), anhydrous ferric chloride (FeCl_3_, 99.99%), sodium salicylate (NaSal, 99%), disodium hydrogen phosphate (Na_2_HPO_4_, 99%), tetraethoxysilane (TEOS), triethanolamine (TEA, 99%), cetyltrimethyl ammonium bromide (CTAB, 99%), bis-(gamma-triethoxy-silyl propyl) tetrasulfide (BTES, 99%), N,n-dimethyl-p-phenylenediamine dihydrochloride (DMPD·2HCl, 99%), 1,2-bis (2-aminophenoxy) -ethane-N,N,N′,N′-tetraacetic acid (BAPTA-AM, 99%), and 3-aminopropyl triethoxysilane (APTES, 99%) were purchased from Aladdin Reagent Co., Ltd. (Shanghai China) 5,5′-Dithiobis (2-nitrobenzoic acid) (DTNB), 5,5-dimethyl-1-pyrrolin-n-oxide (DMPO), methylene blue (MB), fluorescein isothiocyanate (FITC), propidium iodide (PI, 98%), calcein-acetoxymethyl ester (AM, 97%), and 3-(4,5-dimethyl-2-thiazolyl)-2,5-diphenyl-2-H-tetrazolium bromide (MTT, 97%) were purchased from Beyotime Biotechnology Co., Ltd. (Shanghai China)。

### 2.2. Synthesis of DMOS

Firstly, 122 μL of TEA was dispersed in a three-necked flask containing 50 mL of distilled water, which was fixed in an oil bath at 80 °C and stirred by using constant reflux for 0.5 h. Then, 760.0 mg of CTAB and 300.0 mg of NaSal were added to the above solution and continually stirring at 80 °C for 2 h. Next, a mixture of 4 mL TEOS and 3 mL BTES was added to the solution and stirred at 80 °C for 12 h. The products were collected via centrifugation for 10 min at 11,000 r/min (relative centrifugal force: 10,687 g) and washed with ethanol 3 times. Finally, the product was dispersed in 25 mL hydrochloric methanol solution (2M) and stirred at 75 °C for 6 h, where the process was repeated 3 times. The products were dried at room temperature for 12 h, obtaining DMSO NPs. Later, 0.5 g of DMOS powders were dissolved in a three-neck flask containing 30 mL anhydrous toluene, dispersed evenly through the use of ultrasound, and then 0.5 mL APTES was added and fixed in an oil bath at 80 °C for 6 h with constant reflux stirring. The products were collected centrifugation, placed in an oven, and dried at 60 °C for 2 h, obtaining NH_2_-DMOS.

### 2.3. Synthesis of PB@DMOS

Firstly, 0.1 g of NH_2_-DMOS and 160.0 mg of K_3_[Fe(CN)_6_] were added to a three-neck flask containing 10 mL distilled water, dispersing them evenly via ultrasound and adding 50 μL HCl (2M), which was determined as being Solution A. Then, 81.0 of mg FeCl_3_ was dissolved in 1 mL distilled water containing 50 μL HCl (2M) and dispersed evenly via ultrasound, which was determined as being Solution B. Next, solution B was slowly added to solution A, and the solution was stirred at 25 °C for 2 h at constant temperature. The products were washed with distilled water and centrifuged 3 times at the rotational speed of 12,000 r/min (relative centrifugal force: 12,718 g) for 10 min each time. The collected blue solids were placed in the oven, dried at 45 °C for 12 h, and the collected products were ground, finally obtaining PB@DMOS.

### 2.4. Characterization

RigakuD/max-TTR-III diffractometer with Cu Kα radiation under 40 kV was used for testing X-ray diffraction (XRD). A PHILIPS-CM-20-FEG transmission electron microscope (TEM) and energy-dispersive spectrometer (EDS) were used for observing the morphology and element distribution of the materials, with an operating voltage of 200 kV. A Rigaku RAXIS-RAPIDIP X-ray photoelectron spectrometer (XPS) was used for analyzing element valence. A UV-2500 ultraviolet-visible (UV-vis) light absorption spectrometer was selected for testing ultraviolet absorption characteristics. Zetasizer-Nano-ZS was used for measuring surface zeta potential and particle size distribution of the materials via the dynamic light-scattering technique (DLS). A Spotlight 400 infrared spectrometer was used for measuring Fourier transform infrared spectroscopy (FT-IR), where the scanning wavelength range was 500~4000 cm^−1^ and the scanning speed was 16 times/min. An Inf Rec R300SR-HD infrared thermal (IR) imager was used to evaluate the photothermal properties of the materials by irradiating the samples with different power lasers. The images of stained cells and tissue sections were observed using confocal irradiation scanning microscopy (CLSM, TCS SP8). Apoptosis data were characterized flow cytometry (FCM).

### 2.5. H_2_O_2_ Catalytic Performance

Methylene blue (MB) was chosen as an indicator that can react with the hydroxyl radical (•OH) generated by PB@DMOS to form oxide TME (oxTMB), which exhibited a characteristic absorption peak at 664 nm. PB@DMOS (100 μg/mL) were dissolved in 2.5 mL saline phosphate-buffered saline (PBS) buffer solution (pH = 6.5 and 7.4) and reacted with H_2_O_2_ and MB in a 3 mL colorimetric dish. The changes in the absorbance curve over time were measured using a UV-vis spectrophotometer.

### 2.6. GSH Responsive Property

First, 2 mg/mL PB@DMOS were mixed with GSH (10 mM) aqueous solution, followed by adding DTNB (0.2 mM) to detect the -SH group in GSH for different times (0, 1, 2, 4, and 6 h). The absorbance changes at 412 nm were recorded.

### 2.7. Photothermal Properties

The PB@DMOS (140 μg/mL) was prepared and then exposed to an NIR (808 nm) laser for 300 s with different laser power densities (0.50, 0.75, 1.00, and 1.50 W/cm^2^). The temperature changes and infrared thermal images were recorded and captured every 2 min using an IR thermal camera. The photothermal conversion efficiency (*η*) of PB@DMOS was obtained from the change of temperature with time in the cooling stage through the photothermal conversion formula.

### 2.8. In Vitro Evaluation

First, 2 mL of FITC-labeled PB@DMOS solution was incubated with 4T1 cells at different times for evaluation of cellular uptake performance. The L929 and 4T1 cells were seeded in 96-well plates, respectively, where the biocompatibility and cytotoxicity of PB@DMOS were determined using MTT assay. The different concentration of PB@DMOS and different treatment was conducted and incubated with cells, and then 20 μL of MTT solution was added and tested using a microplate reader (UV-vis absorption) at the wavelength of 490 nm. The cytotoxicity of the PB@DMOS is also evaluated, where 5 μL of annexin V-FITC and 5 μL of PI were added to each well for analysis using FCM. AM/PI staining was also conducted to observe live/dead cells to evaluate therapeutic efficiency.

### 2.9. In Vivo Evaluation

The biodistribution of PB@DMOS, the BALB/c mice were injected with 4T1 cells and then injected with PB@DMOS (20 mg/kg) for treatment. The Fe ions concentration in major organs (heart, liver, spleen, lung, and kidneys) and tumors were dissected using ICP-OES. the in vivo anticancer effect, the BALB/c mice injection with 4T1 cells randomly fell into four groups (*n* = 5). The group was set as follows: (1) control, (2) PB, (3) PB@DMOS, and (4) PB@DMOS+808 nm. The mice in the (2)–(4) group were injected intravenously with various samples PB, PB@DMOS, and PB@DMOS, respectively, (20 mg/kg, 0.1 mL). The 808 nm laser irradiation time was 10 min. The tumor volumes and weights of the mice were measured once every two days. After the therapeutic process, the main organs and tumors were collected from groups, where the H&E staining assays were presented to confirm the injury caused by PB@DMOS.

### 2.10. Statistical Analysis

Quantitative data were indicated as mean ± S.D. Student’s *t*-test was used to analyze all experimental data with GraphPad Prism 9.0. Statistical significance was assumed at a value of * *p* < 0.05, ** *p* < 0.01, and *** *p* <0.001.

## 3. Results and Discussion

### 3.1. Morphology and Structure

**Figure 1:**
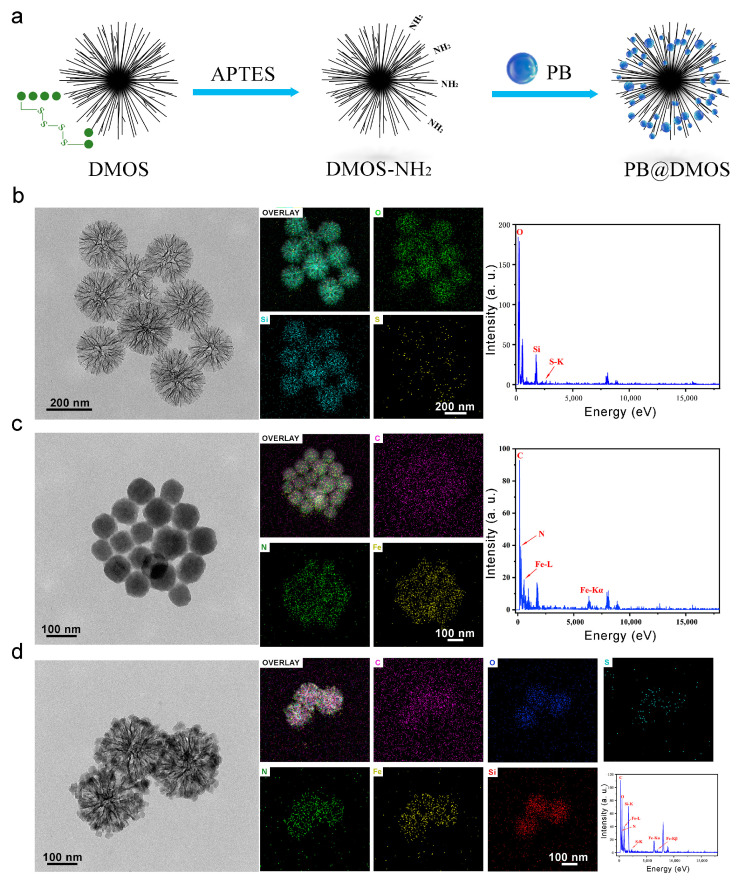
Morphology and structure. (**a**) Schematic illustration of PB@DMOS synthesis. (**b**) TEM image, elemental mapping images, and EDS spectra of DMOS. (**c**) TEM image, elemental mapping images, and EDS spectra of PB. (**d**) TEM image, elemental mapping images, and EDS spectra of PB@DMOS.

As displayed in Figure 1a, the schematic illustration of PB@DMOS synthesis presents that a rationally designed tetra-sulfide-modified DMOS NPs as a silica-based nanocarrier and an H_2_S donor for GSH-triggered H_2_S controllable release then loaded PB NPs (the all-in-one nanoplatforms designated as PB@DMOS) for photothermal-enhanced H_2_S release and amplified ROS generation for efficient combined therapy. The TEM image in Figure 1b shows the well-dispersed DMOS NPs with dendritic spherical morphology, exhibiting a uniform size of around 200 nm. High-angle annular dark field-scanning transmission electron microscopy (HAADF-STEM) images, elemental mapping, and EDS spectrum of the DMOS NPs demonstrate the homogeneous distribution of Si, O, and S in the DMOS NPs, demonstrating the successful doping of S elements. TEM and high-resolution images of PB NPs synthesized via the co-precipitation method using potassium ferricyanide and ferric chloride as raw materials are presented in Figure 1c. The size of PB NPs synthesized directly in an aqueous solution is roughly 40 nm. Due to the unstable structure of PB NPs themselves and the absence of surface functionalization treatment, the PB NPs tend to aggregate and are easily cleared by the phagocytic system of macrophages, are not suitable to be directly used as nanomedical drugs. Fe, C, and N elements can be intuitively seen on the surface of PB NPs by EDS spectra, which exhibit high content and uniform distribution, and their mass fractions were 45.30%, 25.36%, and 29.34%, respectively. TEM and high-resolution images of the PB@DMOS are shown in Figure 1d. It can be seen that the dendritic structure of DMOS after adsorption of PB NPs is not destroyed, and there are a large number of cubic structure PB NPs with limited synthesis in the mesoporous structure, whose particle size is roughly 25 nm, which is about 15 nm smaller than that of PB NPs synthesized directly in water. The average size of the PB@DMOS is around 220 nm, which is slightly larger than DMOS NPs (200 nm). The EDS spectra of the PB@DMOS show that the Fe, C, N, Si, O, and S elements are evenly distributed on the surface of the PB@DMOS, and the loading of PB NPs is good. The above characterization results can confirm the successful synthesis of PB@DMOS.

**Figure 2:**
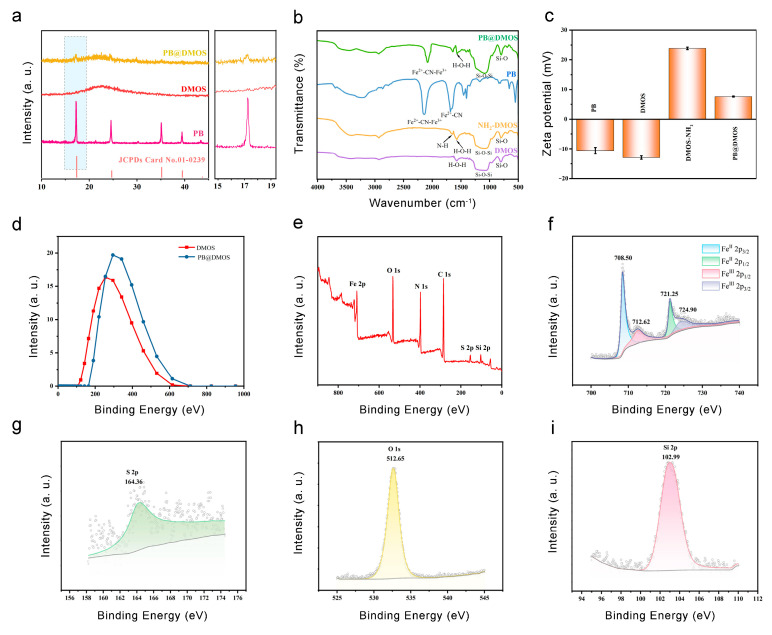
(**a**) XRD spectrum of PB, DMOS, and PB@DMOS. (**b**) FT-IR spectrum of DMOS, NH_2_-DMOS, and PB@DMOS. (**c**) Zeta potential of PB, DMOS, NH_2_-DMOS, and PB@DMOS. (**d**) DLS spectrum of DMOS and PB@DMOS. (**e**) Full XPS spectra of PB@DMOS. (**f**–**i**) High-resolution spectra of Fe, S, O, and Si elements, respectively.

The XRD pattern of the sample (Figure 2a) is in good agreement with the PB standard card (JCPDS No. 01-0239), which proves the synthesis of PB NPs. The XRD spectrum of PB@DMOS is compared with those of DMOS and PB NPs synthesized previously, showing obvious PB NPs and silica diffraction characteristic peaks. The FT-IR spectrum of unmodified DMOS, NH_2_-DMOS obtained via amination, PB NPs, and PB@DMOS were tested, respectively (Figure 2b). The strong and wide absorption band near 1080 cm^−1^ is the Si-O-Si antisymmetric stretching vibration peak. The characteristic peak at 798 cm^−1^ is the Si-O bond symmetric stretching vibration peak. The peak around 1563 cm^−1^ is the H-O-H bending vibration peak of water, and the peak at 955 cm^−1^ belongs to the Si-OH bending vibration absorption peak. After amination, a new characteristic peak appears at 1650 cm^−1^, which is the N-H stretching vibration peak of -NH_2_. Subsequently, PB NPs grow in the mesopores of DMOS. Due to its special metal–organic framework structure, the strong absorption at 2088 cm^−1^ is the characteristic absorption peak of -CN stretching vibration in Fe^2+^-CN-Fe^3+^, while the peak appeared at 1631 cm^−1^ is the cyano vibration peak of CN-Fe^2+^. As shown in Figure 2c, the zeta potential of the synthesized PB NPs is −10.62 mV. After amination modification of DMOS NPs, the measured zeta potential changes from negative (−12.93 mV) to positive (23.87 mV) due to the formation of -NH_3_^+^ by the amino group in water so that PB NPs can be absorbed via electrostatic action. After loading PB NPs, the potential decreases by 16.28 mV but still remains positive (7.59 mV), equipping good biocompatibility. As shown in Figure 2d, the particle size of DMOS in water detected through the use of DLS is 241.7 nm. After loading PB NPs, the particle size of PB@DMOS increases to 323.4 nm, which is slightly larger than the size estimated by the TEM image (around 220 nm). XPS analysis was performed on the PB@DMOS through the use of electron energy spectrometer to characterize the valence information of the surface elements (Figure 2e). Figure 2f is the high-resolution XPS spectrum of Fe 2p in PB@DMOS. The characteristic peaks at the binding energies of 708.50 eV, 712.62 eV, 721.25 eV, and 724.90 eV are derived from the electrons of Fe^Ⅱ^ 2p_3/2_, Fe^Ⅲ^ 2p_1/2_, Fe^Ⅱ^ 2p_1/2_, and Fe^Ⅲ^ 2p_3/2_ in PB NPs, respectively. Figure 2g shows the high-resolution XPS spectrum of S2 2p in PB@DMOS, where the characteristic peak at 164.36 eV binding energy is derived from S2 2p electrons in DMOS. Figure 2h is the high-resolution XPS spectrum of O 1s in PB@DMOS. The characteristic peak at 512.65 eV is derived from the O 1s electron in DMOS. Figure 2i shows the high-resolution XPS spectrum of Si 2p in PB@DMOS. The characteristic peak at 102.99 eV is derived from Si 2p electrons in DMOS.

### 3.2. Photothermal Properties

**Figure 3:**
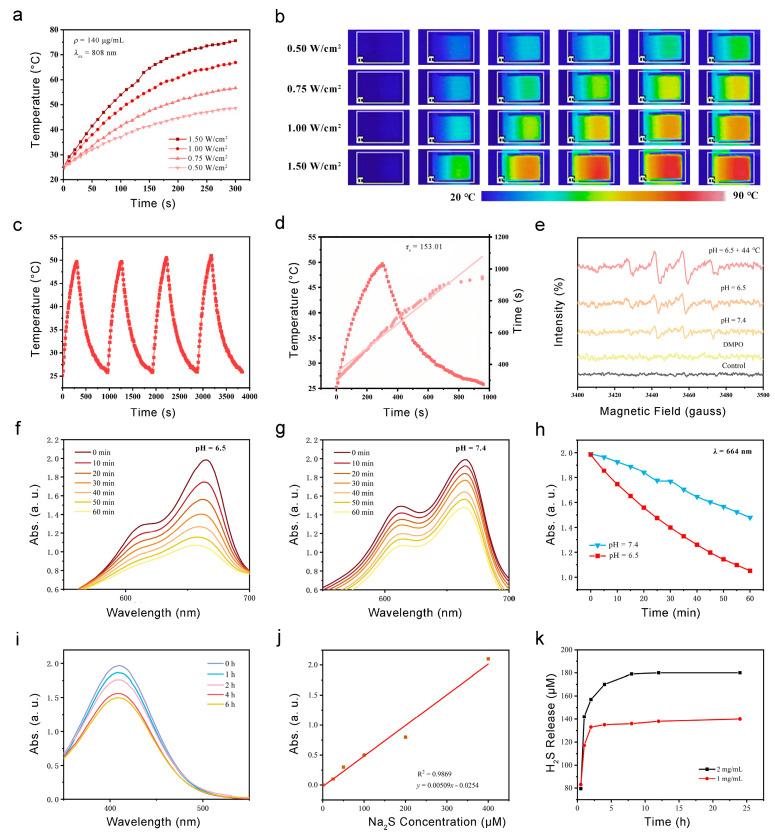
Photothermal properties, ROS generation, and GSH responsive H_2_S release. (**a**) The temperature increase in PB@DMOS under different power densities. (**b**) Infrared thermal images PB@DMOS under different power densities. (**c**) Recycling course of heating and cooling process for four cycles (140 μg/mL, 0.50 W/cm^2^). (**d**) Fitting linear relationship of -ln(θ)-*t* calculating from cooling data. (**e**) ESR spectra for detection of •OH via DMPO under different conditions. (**f**,**g**) UV-vis absorption spectra of MB catalyzed using PB@DMOS group under pH = 6.5 and 7.5. (**h**) The change absorbance with time at 664 nm for oxide MB catalyzed using PB@DMOS group under pH = 6.5 and 7.5. (**i**) UV-vis absorption spectra of GSH consumption using PB@DMOS. (**j**,**k**) H_2_S release properties of PB@DMOS.

Firstly, the heating effect of PB@DMOS is studied. An aqueous solution of 140 μg/mL PB@DMOS solution was prepared, and a NIR (808 nm) laser was used as the external stimulus (excitation light source) to irradiate the sample for 5 min at different laser power density (0.50, 0.75, 1.00, and 1.25 W/cm^2^). The IR camera was used to read and record the temperature rise. The photothermal temperature rise curve is drawn (Figure 3a), and the infrared thermal images of PB@DMOS solution (Figure 3b) are recorded. In the process of laser irradiation, the solution continuously generates heat. According to infrared thermal imaging, it can be intuitively seen that with the increase laser power density, the rate of temperature increase of the PB@DMOS solution becomes higher, and the temperature rises at the same time. After 5 min of laser irradiation, the PB@DMOS solution temperature can reach 75.6 °C under the conditions of 1.50 W/cm^2^ laser power, indicating that PB@DMOS shows excellent photothermal properties under near-infrared light irradiation. The temperatures after 5 min of laser irradiation with powers of 0.50, 0.75, and 1.00 W/cm^2^ are 48.6 °C, 56.7 °C, and 66.9 °C, respectively. The temperature that can effectively kill tumor cells is about 44.0 °C, and a temperature is side effects, such as inflammation, affecting the survival quality of normal cells, therefore 0.50 W/cm^2^ laser power density is selected for the follow-up photothermal performance test.

In order to investigate the photothermal stability of PB@DMOS, a 140 μg/mL PB@DMOS solution was prepared. The solution was irradiated at 0.50 W/cm^2^ laser power for 5 min, then cooled to room temperature naturally, and then turned on to perform laser irradiation. he above steps were repeated three times, the temperature change of the whole process during the recording of the solution was read, and the cyclic rising and cooling curve of the material was drawn (Figure 3c). It can be seen from the experimental results that after four near-infrared light irradiation “switch” cycles, the temperature of the solution remained at about 49.7 °C after 5 min of irradiation, which met the requirements of tumor photothermal treatment. Repeated rising and cooling in a short period of time does not affect the photothermal properties of the material; thereforethe material has good photothermal stability and can achieve repeated treatment. Finally, the photothermal conversion efficiency (*η*) of PB@DMOS is finally calculated, which is determined as 16.0%.

### 3.3. ROS Generation and GSH Responsive H_2_S Release

Fenton catalytic performance is a key index used to evaluate the performance of chemodynamic treatment nanomedical drugs, which directly determines the production rate of •OH and tumor efficacy. The evaluation of Fenton catalytic performance generally uses reactive oxygen species (ROS) detection reagents, fluorescence, ultraviolet spectrometers, and other means to determine the •OH generated via the Fenton reaction. At first, DMPO, the most commonly used spin-trapping agent in the study of free radicals, is used to explore the Fenton reaction between PB@DMOS and H_2_O_2_ through its unique electron spin resonance (ESR) spectra. PB@DMOS and H_2_O_2_ were added successively to PBS buffers with pH values of 6.5 and 7.5 under room temperature and pH 6.5 + 44 °C condition characteristic signal peaks of 1:2:2:1 appeared in ESR spectra (Figure 3e), indicating that under conditions of weak acidity and high temperature (44 °C), PB@DMOS can react with H_2_O_2_ to produce •OH. Moreover, the characteristic peak intensity is the highest at 44 °C and under pH = 6.5 conditions; therefore, the weak acidity and high-temperature environment of tumor hyperthermia can enhance the Fenton reactivity of the material and H_2_O_2_. UV-vis spectrophotometry refers to the method of quantitative or qualitative analysis of substances based on the absorption of ultraviolet and visible light via the measured substance molecules. Because •OH itself cannot be detected through the use of spectrophotometry, methylene blue (MB) is selected as the detection reagent, and •OH can oxidize MB from blue to colorless. As shown in Figure 3f–h, in aweakly acidic environment (pH = 6.5), the H_2_O_2_ catalytic efficiency of PB@DMOS is higher, and the H_2_O_2_ consumption rate reached 47.0% after 60 min of reaction, which is twice as fast as that in a weakly alkaline environment (pH = 7.5, 25.6%). According to the change curve of the absorbance peak with the reaction time, it can be seen that the catalytic reaction speed is first fast and then slow under the weakly acidic condition, which is caused by the gradual reduction H_2_O_2_ substrate concentration with the reaction, while the catalytic reaction rate is slow and unstable under weakly alkaline conditions. Therefore, the weak acidity of the tumor microenvironment is more suitable for the smooth and efficient H_2_O_2_ catalytic process of PB@DMOS.

GSH can react with DTNB to produce yellow TNB and oxidized glutathione, which have characteristic absorption peaks at 410 nm. Thus, 2 mg/mL PB@DMOS was mixed with GSH (10.0 mM) aqueous solution, and then DTNB (0.2 mM) was addedthe for the 6 h reaction. The absorbance changes in terms the solution at different times (0, 1, 2, 4, and 6 h) during the reaction were measured and recorded through the use of a UV-vis absorption spectrometer (Figure 3i). The results show that PB@DMOS can consume GSH, which further confirms the successful doping of tetrasulfide bond. The reaction can be carried out more gently for a long time, more than 6 h, and the reaction a gas with a pungent odor. According to the degree of decline in terms of absorbance, it can be seen that reaction speed does not change much in the first 4 h, and the speed begins to slow down 4–6 h. After 6 h, the absorbance decreased by 23.8%, and the material had good GSH consumption capacity.

To quantitatively determine the formation of H_2_S, different concentrations of PB@DMOS (1 and 2 mg/mL) were dispersed in GSH aqueous solution (10 mM, 15 mL), which was stirred for different amounts of time (0.2, 0.5, 1, 3, 6, 12, and 24 h). The supernatant was collected and mixed with a mixture of zinc acetate and sodium acetate (mass ratio 4:1, 15 mL). Then, 16.0 mg of DMPD·2HCl and 27.0 mg of FeCl_3_ were added to form MB. The absorbance at 660 nm was measured using a UV-vis absorption spectrometer, and the H_2_S release concentration was calculated using the equation fitted with the Na_2_S standard curve (Figure 3j). The experimental results show that the two concentrations of PB@DMOS solutions have GSH-responsive H_2_S release ability. The H_2_S release concentration of 1 mg/mL PB@DMOS solution after reaction for 24 h is 143.98 μM, and the release concentration of 2 mg/mL PB@DMOS solution was 181.02 μM. The release rate is fast in the first 4 h and gradually flattens over time, but H_2_S gas can still be released. The high release rate in the early stage ensures that H_2_S gas can reach the required concentration level of gas therapy and avoid the tumor-promoting effect caused by low-concentration gas (Figure 3k).

### 3.4. In Vitro Anti-Tumor Performance

**Figure 4:**
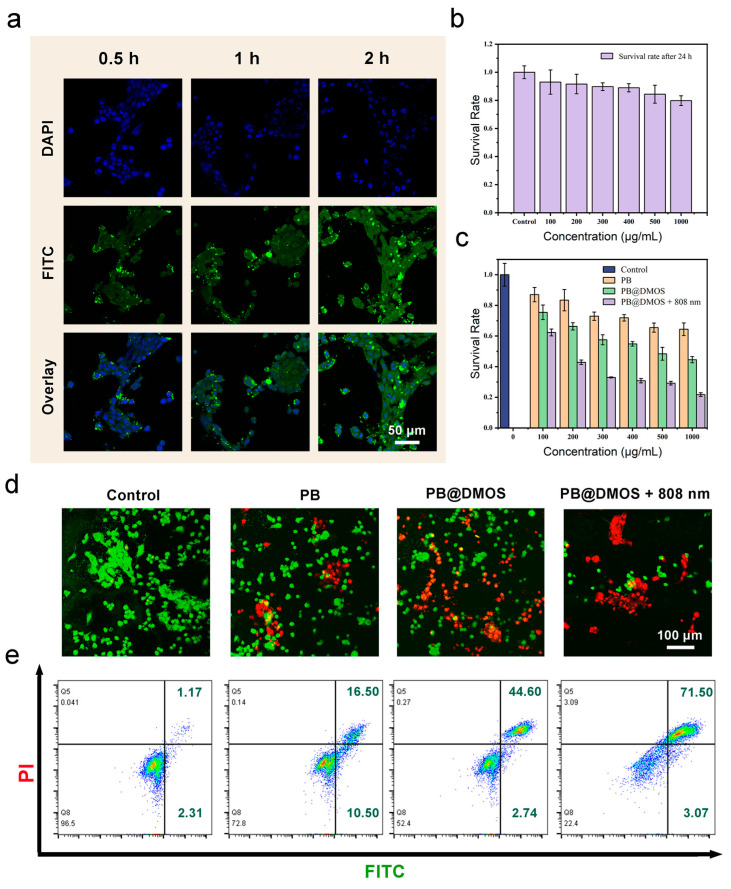
In vitro anti-tumor performance. (**a**) 4T1 cells uptake behavior of FITC-labeled PB@DMOS. (**b**,**c**) Cell viabilities of PB@DMOS treated L929 cells at different concentrations and 4T1 cells at different treatment groups with different concentrations. (**d**) Live/dead staining of 4T1 cells with different treatments. (**e**) Flow cytometry analysis of apoptosis in 4T1 cells staining with PI/Annexin FITC after treatment.

Relation to advanced GSH-GSH-responsive consumption, H_2_S controllable release, and the efficient ROS generation of PB@DMOS, the in vitro anti-tumor performance of PB@DMOS is further investigated. Endocytosis is the primary way that nanomaterials areingested by cells. The phagocytosis of PB@DMOS was accomplished by tracking the fluorescence intensity of the fluorescein isothiocyanate (FITC)-labeled PB@DMOS within the cells CLSM at different incubation times. The green fluorescence signal intensity of FITC-labeled PB@DMOS is enhanced with prolonged time, demonstrating PB@DMOS isby 4T1 cells after 2 h of incubation (Figure 4a). The MTT assay is adopted to detect the biocompatibility and cytotoxicity of PB@DMOS on fibroblast cells (L929 cells) and mouse breast cancer cell lines (4T1 cells). As shown in Figure 4b, PB@DMOS shows negligible toxicity to normal cells at different concentrations for 24 h the survival rate of cells is still over 80% until the concentration of PB@DMOS is 1000 μg/mL. Figure 4c shows the toxicity of PB@DMOS to 4T1 cells concentration-dependent under different treatment condition, which is related to H_2_S release, the H_2_O_2_ catalyst, and the photothermal effect of PB@DMOS nanoalloys when stimulating the metabolism of 4T1 cells, in the PB@DMOS+808 nm group (1000 μg/mL), the survival rate of 4T1 cells is around 20%. In order to distinguish the live cells from dead cells visually, co-staining with Calcein-AM/PI is conducted in different groups, as follows: (1) control, (2) PB, (3) PB@DMOS, and (4) PB@DMOS+808 nm. As presented in Figure 4d, the control group does not exhibit significant cell damage. However, a minority of dead cells are shown in the PB groups, which could be attributed to the H_2_O_2_-catalyzed effect of PB NPs. A majority of dead cells are observed in the PB@DMOS group, which is due to the additional H_2_S release properties of PB@DMOS. The PB@DMOS+808 nm group exhibits the highest number of dead cells, which is consistent with GSH responsive consumption, H_2_S controllable release, and ROS efficient generation of PB@DMOS performance observed in the previous experiments. The annexin V-FITC/PI apoptosis detection kit was utilized for binding to associated proteins within cells via flow cytometry to assess early and late apoptotic cells (Figure 4e), further demonstrating the above results. The 4T1 cells in the PB@DMOS+808 nm group undergo apoptosis, and the proportion of apoptotic cells in the PB@DMOS+808 nm group is up to 74.6% in the Q2 + Q3 region.

### 3.5. In Vivo Anti-Tumor Performance

**Figure 5:**
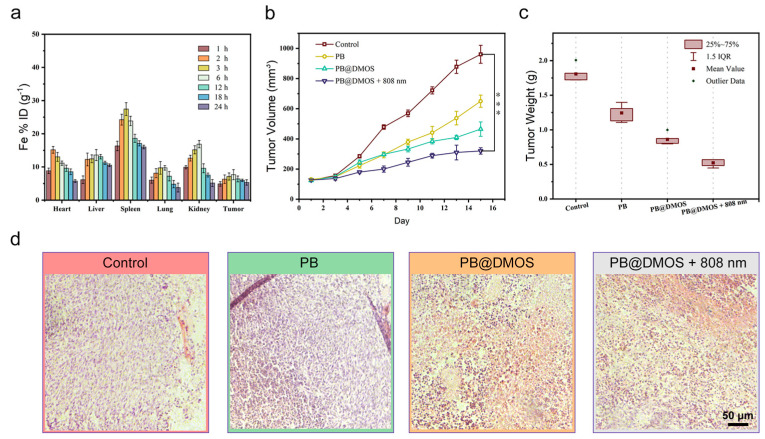
In vivo anti-tumor performance. (**a**) Biodistribution of Fe in main organs and tumors of mice at varied time points after PB@DMOS solution administration. (**b**) Tumor volume growth curves of mice in all groups after different treatments. Data presented as mean ± S.D. (*n* = 5). (**c**) Tumor weight of mice in all groups after different treatments. Data presented as mean ± S.D. (*n* = 5). (**d**) H&Estaining assay of tumor tissues after different treatments.

For in vivo anti-tumor performance evaluation of PB@DMOS, 4T1 tumor-bearing mice were established to evaluate the therapeutic efficacy of PB@DMOS. Firstly, the biodistribution of PB@DMOS in major organs and tumors was investigated. As presented in Figure 5a, PB@DMOS could be efficiently accumulated at tumor sites due to the enhanced permeability and retention (EPR) effect. Then, the 4T1 tumor-bearing mice were randomly divided into four groups as follows: (1) control, (2) PB, (3) PB@DMOS, and (4) PB@DMOS+808 nm. As shown in Figure 5b, tumor growth in control groups experienced no change. At the same time, tumor growth in PB and PB@DMOS groups was slightly inhibited after the treatment process. Significantly, tumor growth in the PB@DMOS+808 nm group is markedly suppressed. In addition, the tumor weight in different treatments changes apparently (Figure 5c). H&E staining images of major organs from the mice after the therapeutic process (Figure 5d) show no injury, indicating that the PB@DMOS possesses negligible side effects during the whole treatment.

## 4. Conclusions

In summary, a GSH-responsive/photothermal-enhanced nanoplatform with tetra-sulfide-incorporated DMOS NPs as the nanocarrier and H_2_S donor to load functional PB NPs was successfully constructed. As tetra-sulfide bonds were sensitive to reductive GSH, the DMOS NPs achieved GSH depletion and controllable H_2_S release for gas therapy. The temperature increase and oxidative stress caused by the PB NP loading promoted H_2_S release and amplified the ROS effect. After in vitro and in vivo applications, PB@DMOS effectively accumulated at the tumor site, resulting in a collective anti-tumor performance owing to GSH consumption, H_2_S release, ROS generation, and temperature increase. Our therapeutic gas strategy may provide a new opportunity for combined tumor therapy.

## Data Availability

The data presented in this study are available on request from the corresponding author. The data are not publicly available due to innovation.
